# Supplementary material for: Trends and Determinants of Full Immunisation among Children Aged 12–23 Months: Analysis of Pooled Data from Mozambican Household Surveys between 1997 and 2015
Source: Int J Environ Res Public Health. 2023 Jan 31;20(3):2558. doi: 10.3390/ijerph20032558 (PMC9915409; doi:10.3390/ijerph20032558)
Supplement: Supplementary file 1 [file ijerph-20-02558-s001.zip › ijerph-2051027-supplementary.pdf]

Trends and Determinants of Full Immunisation among Children Aged 12–23 Months: Analysis of Pooled Data from Mozambican Household Surveys between 1997 and 2015

Table S1: Projected Mozambique Population for 2020 (based on Census 2017) per province[1]

Province	Total			Under 5 years						Between 12 and 23 months*		
	Total	Men	Women	Total		Men		Women		Total	Men	Women
				N	%^	N	%^	N	%^			
Cabo Delgado	2,525,416	1,226,849	1,298,567	393,527	15.6	197,678	16.1	195,849	15.1	68,145	34,261	33,884
Gaza	1,445,896	657,230	788,666	183,099	12.7	91,339	13.9	91,760	11.6	31,706	15,831	15,876
Inhambane	1,531,959	707,423	824,536	181,756	11.9	91,264	12.9	90,492	11.0	31,474	15,818	15,656
Manica	2,114,507	1,017,547	1,096,960	368,376	17.4	184,872	18.2	183,504	16.7	63,790	32,041	31,749
Maputo City	1,124,988	546,311	578,677	113,724	10.1	57,531	10.5	56,193	9.7	19,693	9,971	9,722
Maputo Province	2,216,460	1,066,608	1,149,852	269,899	12.2	136,163	12.8	133,736	11.6	46,737	23,599	23,138
Nampula	6,183,863	3,014,653	3,169,210	973,455	15.7	488,434	16.2	485,021	15.3	168,569	84,654	83,915
Niassa	1,998,266	969,134	1,029,132	335,048	16.8	168,486	17.4	166,562	16.2	58,019	29,202	28,817
Sofala	2,457,828	1,194,168	1,263,660	396,694	16.1	199,416	16.7	197,278	15.6	68,694	34,562	34,132
Tete	2,900,213	1,423,794	1,476,419	447,678	15.4	224,332	15.8	223,346	15.1	77,523	38,881	38,642
Zambezia	5,567,252	2,677,479	2,889,773	947,640	17.0	475,795	17.8	471,845	16.3	164,099	82,463	81,635
Mozambique	30,066,648	4,501,196	15,565,452	4,610,896	15.3	2,315,310	16.0	2,295,586	14.7	798,449	401,283	397,166

^ Proportion of under-5 year among the total population

* The official Census projections includes the 12 to 23 months, the interest group to assess the full immunization coverage, as part of the whole under-5. We use World Bank interpolated proportion (0.173) of under-5 that are within 12 to 36 months of life.

Table S2: Descriptive and bivariate regression analysis of children aged 12-23 months, 1997 survey.

Characteristic	Total	Not immunised/Partially	Fully immunised			Crude		
	**N (%)**	N (%)	**N (%)**	**Coverage (%)**	**95%CI**	**OR**	**95% CI**	***p*-value**
Total of children enrolled	1240 (100.0)	647 (100.0)	593 (100.0)	47.9	42.8-52.9			
**Mother's age group**								0.197
15-24	472 (38.0)	225 (34.8)	247 (41.6)	52.3	46.4-58.2	1.00	-	
25-34	524 (42.3)	286 (44.2)	238 (40.2)	45.5	38.4-52.8	0.76	0.55-1.05	0.093
≥ 35	244 (19.7)	136 (21.0)	108 (18.2)	44.3	31.0-58.4	0.73	0.39-1.36	0.316
**Mother's education**								<0.001
Illitarate	446 (35.9)	309 (47.8)	137 (23.1)	30.7	24.8-37.4	1.00	-	
Primary	739 (59.6)	332 (51.4)	407 (68.6)	55.1	49.4-60.6	2.76	2.06-3.70	<0.001
Secondary/above	55 (4.5)	6 (0.9)	50 (8.4)	89.7	77.3-95.8	19.75	7.44-52.42	<0.001
**Mother's marital status**								0.001
Single/Never in union	47 (3.8)	9 (1.5)	37 (6.3)	79.8	65.2-89.2	4.50	2.07-9.76	<0.001
Married/Cohabitation	1088 (87.8)	580 (89.7)	508 (85.7)	46.7	40.6-52.9	1.00	-	
Divorced/Separated/Widowed	105 (8.5)	57 (8.9)	48 (8.0)	45.4	28.1-63.8	0.95	0.39-2.29	0.903

**Mother's occupation**								<0.001
Unemployed	467 (37.7)	174 (27.0)	292 (49.5)	62.6	54.4-70.2	1.00	-	
Employed	771 (62.3)	472 (73.0)	299 (50.5)	38.8	32.5-45.4	0.38	0.24-0.59	<0.001
**Area of residence**								<0.001
Urban	280 (22.6)	41 (6.4)	238 (40.2)	85.2	80.5-88.9	9.81	6.28-15.31	<0.001
Rural	960 (77.4)	605 (93.6)	355 (59.8)	37.0	30.3-44.2	1.00	-	
**Wealth index**								<0.001
Poorest	203 (16.7)	162 (25.5)	41 (7.2)	20.4	8.8-40.4	1.00	-	
Poorer	216 (17.8)	157 (24.7)	59 (10.2)	27.4	17.7-39.9	1.48	0.43-5.07	0.534
Middle	288 (23.7)	185 (29.2)	103 (17.8)	35.7	24.6-48.6	2.17	0.71-6.67	0.176
Richer	248 (20.4)	80 (12.6)	168 (29.0)	67.8	57.3-76.7	8.21	2.71-24.89	<0.001
Richest	259 (21.3)	51 (8.0)	208 (35.9)	80.4	72.8-86.3	16.03	5.78-44.46	<0.001
**Religion**								0.015
Catholic	378 (30.6)	217 (33.7)	161 (27.3)	42.7	34.1-51.8	1.00	-	
Islamic	190 (15.4)	114 (17.7)	77 (13.0)	40.3	26.7-55.5	0.91	0.45-1.82	0.781
Protestant	384 (31.2)	154 (24.0)	230 (39.0)	59.8	52.3-66.9	2.00	1.23-3.24	0.005
Others	281 (22.8)	159 (24.7)	122 (20.8)	43.6	31.7-56.2	1.04	0.55-1.96	0.910
**Antenatal visits**								<0.001
No visits	258 (23.0)	247 (41.4)	10 (2.0)	4.0	2.3-7.0	1.00	-	
1 to 3	317 (28.3)	148 (24.8)	169 (32.4)	53.3	44.3-62.1	27.14	13.60-54.17	<0.001

≥ 4	545 (48.7)	202 (33.8)	343 (65.6)	62.9	56.8-68.6	40.26	21.55-75.23	<0.001
**Place of delivery**								<0.001
At home/other	630 (51.1)	438 (68.3)	192 (32.5)	30.5	22.3-40.0	1.00	-	
Institutional	603 (48.9)	203 (31.7)	400 (67.5)	66.3	58.9-72.9	4.49	2.34-8.61	<0.001
**Sex of the child**								0.978
Male	635 (51.2)	332 (51.3)	303 (51.1)	47.8	40.1-55.6	0.99	0.56-1.75	0.978
Female	605 (48.8)	315 (48.7)	290 (48.9)	48.0	38.6-57.5	1.00	-	
**Birth order**								0.060
1	226 (18.3)	97 (15.0)	129 (21.8)	57.1	48.4-65.4	1.65	1.08-2.51	0.020
2 to 3	472 (38.1)	250 (38.6)	222 (37.5)	47.1	39.9-54.4	1.10	0.77-1.58	0.590
≥ 4	542 (43.7)	300 (46.3)	242 (40.7)	44.7	37.8-51.7	1.00	-	
**Has a health card**								<0.001
No card	244 (19.7)	243 (37.8)	1 (0.1)	0.3	0.1-0.9	1.00	-	
Yes seen	821 (66.4)	243 (37.8)	578 (97.3)	70.3	64.7-75.4	763,56	271.07-2150.81	<0.001
Yes not seen	172 (13.9)	156 (24.3)	15 (2.5)	8.8	2.7-25.3	31.08	6.05-159.76	<0.001

Table S3: Descriptive and bivariate regression analysis of children aged 12-23 months, 2003 survey.

**Characteristic**	**Total**	**Not immunised/Partially**	**Fully immunised**			**Crude**		
	**N (%)**	N (%)	**N (%)**	**Coverage (%)**	**95%CI**	**OR**	**95% CI**	***p*-value**
Total of children enrolled	1931 (100.0)	699 (100.0)	1232 (100.0)	63.8	60.6-66.9			
**Mother's age group**								0.015
15-24	761 (39.4)	238 (34.1)	523 (42.4)	68.7	64.5-72.6	1.00	-	
25-34	798 (41.3)	317 (45.3)	482 (39.1)	60.3	55.5-65.0	0.69	0.53-0.89	0.006
≥ 35	372 (19.3)	144 (20.6)	228 (18.5)	61.3	55.1-67.1	0.72	0.53-0.97	0.033
**Mother's education**								<0.001
Illitarate	873 (45.2)	444 (63.6)	429 (53.9)	49.1	44.4-53.9	1.00	-	
Primary	977 (50.6)	253 (36.2)	724 (58.8)	74.1	70.6-77.4	2.96	2.30-3.81	<0.001
Secondary/above	81 (4.2)	2 (0.3)	79 (6.4)	97.7	89.7-99.5	44.74	8.88-225.40	<0.001
**Mother's marital status**								0.030
Single	87 (4.5)	19 (2.7)	68 (5.5)	78.3	63.9-88.1	2.19	1.04-4.63	0.039
Married/Cohabitation	1640 (84.9)	620 (88.7)	1020 (82.8)	62.2	58.7-65.6	1.00	-	
Divorced/Separated/Widowed	204 (10.6)	60 (8.6)	144 (11.7)	70.6	62.8-77.3	1.46	1.01-2.09	0.042

**Mother's occupation**								<0.001
Unemployed	418 (21.6)	86 (12.3)	332 (26.9)	79.4	73.5-84.3	1.00	-	
Employed	1513 (78.4)	613 (87.7)	900 (73.1)	59.5	55.8-63.1	0.38	0.26-0.55	<0.001
**Area of residence**								<0.001
Urban	575 (29.7)	111 (15.9)	464 (37.6)	80.7	74.8-85.4	3.19	2.20-4.64	<0.001
Rural	1357 (70.3)	588 (84.1)	769 (62.4)	56.7	52.8-66.3	1.00	-	
**Wealth index**								<0.001
Poorest	508 (26.3)	276 (39.5)	232 (18.9)	45.7	39.6-52.0	1.00	-	
Poorer	362 (18.7)	167 (23.8)	195 (15.8)	53.9	46.6-61.1	1.39	0.95-2.04	0.090
Middle	415 (21.5)	157 (22.5)	258 (20.9)	62.1	55.7-68.1	1.95	1.39-2.73	<0.001
Richer	329 (17.0)	70 (10.0)	259 (21.0)	78.7	73.7-83.1	4.39	3.02-6.39	<0.001
Richest	317 (16.4)	29 (4.1)	288 (24.4)	90.9	85.8-94.2	11.79	6.60-21.06	<0.001
**Religion**								<0.001
Catholic	541 (33.4)	208 (36.0)	333 (32.0)	61.6	55.6-67.3	1.00		
Islamic	344 (21.3)	148 (25.7)	196 (18.8)	57.0	50.1-63.6	0.83	0.57-1.21	0.319
Protestant	528 (32.7)	178 (30.9)	350 (33.7)	66.3	60.7-71.4	1.23	0.88-1.70	0.228
Other	204 (12.6)	42 (7.3)	161 (15.5)	79.2	73.0-84.4	2.38	1.56-3.63	<0.001
**Antenatal visits**								<0.001
No visits	257 (13.8)	205 (30.1)	52 (4.4)	20.4	14.1-28.4	1.00	-	
1 to 3	564 (30.3)	213 (31.4)	351 (29.7)	62.2	56.6-67.4	6.43	3.95-10.46	<0.001
≥ 4	1041 (55.9)	261 (38.5)	780 (65.9)	74.9	71.1-78.4	11.67	7.19-18.94	<0.001

**Place of delivery**								<0.001
At home/other	948 (49.1)	484 (69.2)	465 (37.7)	49.0	44.7-53.3	1.00	-	
Institutional	983 (50.9)	215 (30.8)	768 (62.3)	78.1	74.3-81.5	3.72	2.80-4.93	<0.001
**Sex of the child**								0.791
Male	997 (51.6)	357 (51.1)	640 (51.9)	64.2	60.3-67.9	1.03	0.82-1.30	0.791
Female	934 (48.4)	341 (48.9)	592 (48.1)	63.4	58.9-67.8	1.00	-	
**Birth order**								<0.001
1	375 (19.4)	99 (14.2)	276 (22.4)	73.6	68.9-78.5	2.05	1.52-2.76	<0.001
2 to 3	649 (33.6)	215 (30.8)	434 (35.2)	66.8	62.2-71.2	1.48	1.15-1.92	0.003
≥ 4	907 (46.9)	384 (55.0)	522 (42.4)	57.6	53.0-62.0	1.00	-	
**Has a health card**								<0.001
No card	250 (13.0)	236 (33.8)	14 (1.1)	5.6	2.8-11.0	1.00	-	
Yes seen	1507 (78.1)	338 (48.4)	1170 (94.9)	77.6	74.2-80.7	58.20	27.66-122.48	<0.001
Yes not seen	173 (8.9)	124 (17.8)	48 (3.9)	28.1	19.7-38.4	6.56	3.01-14.30	<0.001

Table S4: Descriptive and bivariate logistic regression analysis of children aged 12-23 months, 2011 survey.

Characteristic	Total	Not immunised/Partially	Fully immunised			Crude		
	**N (%)**	N (%)	**N (%)**	**Coverage (%)**	**95%CI**	**OR**	**95% CI**	***p*-value**
Total of children enrolled	2325 (100.0)	821 (100.0)	1504 (100.0)	64.7	61.3-67.9			
**Mother's age group**								0.041
15-24	887 (38.1)	295 (36.0)	591 (39.3)	66.7	61.9-71.2	1.00	-	
25-34	990 (42.6)	339 (41.3)	651 (43.3)	65.8	61.3-70.0	0.96	0.74-1.24	0.755
≥ 35	448 (19.3)	187 (22.8)	261 (17.4)	58.3	52.1-64.2	0.69	0.52-0.93	0.015
**Mother's education**								0.001
Illitarate	808 (34.8)	330 (40.2)	478 (31.8)	59.1	53.6-64.4	1.00	-	
Primary	1217 (52.4)	418 (50.9)	799 (53.1)	65.6	61.7-69.4	1.32	1.04-1.68	0.024
Secondary/above	299 (12.9)	73 (8.9)	227 (15.1)	75.7	68.9-81.4	2.15	1.44-3.22	<0.001
**Mother's marital status**								0.304
Single/Never in union	105 (4.5)	30 (3.6)	76 (5.0)	71.8	62.4-79.6	1.42	0.91-2.21	0.123
Married/Cohabitation	2000 (86.0)	716 (87.1)	1285 (85.4)	64.2	60.7-67.6	1.00	-	

Divorced/Separated/Widowed	179 (7.7)	60 (7.3)	119 (7.9)	66.5	57.0-74.8	1.05	0.73-1.51	0.784
**Mother's occupation**								0.920
Unemployed	1263 (54.5)	448 (54.7)	815 (54.4)	54.4	51.1-57.7	1.00	-	
Employed	1054 (45.5)	371 (45.3)	683 (45.6)	64.8	60.0-69.3	1.01	0.80-1.28	0.920
**Area of residence**								<0.001
Urban	632 (27.2)	156 (19.0)	477 (31.7)	75.4	71.2-79.1	1.98	1.50-2.62	<0.001
Rural	1692 (72.8)	665 (81.0)	1027 (68.3)	60.7	56.4-64.8	1.00	-	
**Wealth index**								<0.001
Poorest	517 (22.3)	234 (28.6)	283 (18.8)	54.7	47.9-61.3	1.00	-	
Poorer	565 (24.3)	238 (29.0)	327 (21.7)	57.8	51.9-63.5	1.14	0.83-1.55	0.419
Middle	460 (19.8)	154 (18.7)	306 (20.4)	66.6	60.0-72.5	1.65	1.17-2.34	0.005
Richer	432 (18.6)	112 (13.6)	321 (21.3)	74.2	69.4-78.4	2.38	1.66-3.41	<0.001
Richest	351 (15.1)	83 (10.2)	267 (17.8)	76.2	70.4-81.2	2.66	1.78-3.96	<0.001
**Religion**								0.127
Catholic	703 (30.2)	272 (33.2)	431 (28.6)	61.2	55.1-67.1	1.00	-	
Islamic	384 (16.5)	120 (14.6)	265 (17.6)	68.9	61.6-75.3	1.39	0.95-2.01	0.088
Protestants	548 (23.6)	175 (21.3)	373 (24.8)	68.1	62.4-73.3	1.35	0.98-1.88	0.071
Others	689 (29.6)	254 (31.0)	435 (28.9)	63.1	58.0-67.9	1.08	0.79-1.48	0.620
**Antenatal visits**								<0.001
No visits	183 (8.3)	141 (18.1)	42 (2.9)	22.9	15.9-31.7	1.00	-	

1 to 3	926 (41.7)	292 (37.3)	634 (44.1)	68.5	64.0-72.7	7.33	4.58-11.74	<0.001
≥ 4	1108 (50.0)	348 (44.6)	760 (52.9)	68.6	64.3-72.6	7.36	4.58-11.83	<0.001
**Place of delivery**								<0.001
At home/other	1069 (46.5)	502 (62.2)	567 (38.0)	53.0	48.0-58.0	1.00	-	
Institutional	1231 (53.5)	305 (37.8)	927 (62.0)	75.2	71.6-78.6	2.69	2.06-3.51	<0.001
**Sex of the child**								0.513
Male	1137 (48.9)	411 (50.1)	726 (48.3)	63.8	59.7-67.8	0.93	0.75-1.15	0.513
Female	1187 (51.1)	410 (49.9)	777 (51.7)	65.5	61.3-69.5	1.00	-	
**Birth order**								0.008
1	515 (22.1)	165 (20.1)	350 (23.3)	68.0	62.2-73.3	1.38	1.05-1.81	0.022
2 to 3	779 (33.5)	251 (30.6)	528 (35.1)	67.8	63.0-72.2	1.36	1.08-1.72	0.010
≥ 4	1031 (44.3)	405 (49.4)	626 (41.6)	60.7	56.3-64.9	1.00	-	
**Has a health card**								<0.001
No card	250 (10.8)	226 (27.5)	24 (1.6)	9.7	6.3-14.7	1.00	-	
Yes seen	1931 (83.1)	467 (56.8)	1464 (97.4)	75.8	72.6-78.8	29.19	17.89-47.66	<0.001
Yes not seen	144 (6.2)	129 (15.7)	15 (1.0)	10.5	6.1-17.5	1.09	0.53-2.26	0.810

Table S5: Descriptive and bivariate regression analysis of children aged 12-23 months, 2015 survey.

**Characteristic**	**Total**	**Not immunised/Partially**	**Fully immunised**			**Crude**		
	**N (%)**	N (%)		**Coverage (%)**	**95%CI**	**OR**	**95% CI**	***p*-value**
Total of children enrolled	1131 (100.0)	379 (100.0)	752 (100.0)	66.5	62.1-70.6			
**Mother's age group**								0.970
15-24	519 (45.9)	175 (46.2)	345 (45.8)	66.4	61.4-73.0	1.00	-	
25-34	406 (35.9)	134 (35.3)	273 (36.2)	67.1	60.9-72.7	1.03	0.73-1.47	0.850
≥ 35	205 (18.1)	70 (18.5)	135 (17.9)	65.8	56.4-74.2	0.98	0.66-1.45	0.906
**Mother's education**								<0.001
Illitarate	316 (28.0)	147 (38.9)	169 (22.5)	53.5	46.3-60.5	1.00	-	
Primary	644 (57.0)	206 (54.5)	438 (58.2)	68.0	61.2-74.1	1.83	1.21-2.82	0.005
Secondary/above	170 (15.0)	25 (6.6)	145 (19.3)	85.3	78.5-90.3	5.07	3.07-8.38	<0.001
**Mother's marital status**								0.158
Single/Never in union	73 (6.5)	20 (5.3)	53 (7.1)	72.5	58.6-83.0	1.27	0.66-2.46	0.467
Married/Cohabitation	871 (77.0)	284 (75.0)	587 (78.0)	67.4	62.6-71.8	1.00	-	

Divorced/Separated/Widowed	187 (16.5)	74 (19.6)	112 (14.9)	60.2	50.4-69.2	0.73	0.48-1.11	0.136
**Mother's occupation**								0.555
Unemployed	637 (56.4)	207 (54.8)	430 (57.2)	67.4	61.5-72.8	1.00	-	
Employed	493(43.6)	171 (45.2)	322 (42.8)	65.3	59.7-70.5	0.91	0.66-1.25	0.555
**Area of residence**								0.001
Urban	287 (25.4)	63 (16.8)	223 (29.7)	77.9	71.0-83.5	2.09	1.37-3.20	0.001
Rural	844 (74.6)	315 (83.2)	529 (70.3)	62.7	57.4-67.7	1.00	-	
**Wealth index**								<0.001
Poorest	278 (24.6)	124 (32.9)	154 (20.4)	55.3	47.6-62.7	1.00	-	
Poorer	262 (23.2)	118 (31.2)	144 (19.2)	54.9	45.1-64.4	0.99	0.63-1.53	0.951
Middle	217 (19.2)	71 (18.9)	146 (19.4)	67.1	58.0-75.1	1.65	0.99-2.75	0.054
Richer	216 (19.1)	41 (10.8)	175 (23.2)	81.0	71.4-87.9	3.45	1.88-6.32	<0.001
Richest	158 (13.9)	23 (6.2)	134 (17.8)	85.1	80.0-89.1	4.63	2.89-7.42	<0.001
**Religion**								0.028
Catholic	334 (29.6)	135 (35.7)	199 (26.5)	59.6	51.2-67.4	1.00	-	
Islamic	224 (19.8)	52 (13.7)	172 (22.9)	76.9	67.9-83.9	2.26	1.31-3.88	0.003
Protestant	302 (26.8)	95 (25.0)	208 (27.7)	68.6	61.3-75.2	1.49	0.94-2.35	0.089
Others	269 (23.8)	97 (25.6)	172 (22.9)	63.9	56.4-70.7	1.20	0.77-1.86	0.412
**Antenatal visits**								<0.001
No visits	77 (7.1)	55 (15.0)	22 (3.1)	29.0	17.4-44.2	1.00	-	

1 to 3	382 (35.5)	150 (41.0)	232 (32.7)	60.9	53.2-68.0	3.80	1.95-7.41	<0.001
≥ 4	616 (57.3)	161 (44.1)	456 (64.1)	73.9	68.9-78.4	6.93	3.40-14.12	<0.001
**Place of delivery**								<0.001
At home/other	316 (27.9)	161 (42.6)	155 (20.6)	49.0	40.4-57.7	1.00	-	
Institutional	815 (72.1)	217 (57.4)	597 ((79.4)	73.3	68.6-77.5	2.86	1.87-4.38	<0.001
**Sex of the child**								0.266
Male	555 (49.1)	173 (45.8)	382 (50.8)	68.8	62.8-74.2	1.23	0.86-1.75	0.266
Female	575 (50.9)	205 (54.2)	370 (49.2)	64.3	58.2-70.0	1.00	-	
**Birth order**								0.461
1	265 (23.4)	86 (22.8)	178 (23.7)	67.4	59.0-74.8	1.17	0.80-1.68	0.422
2 to 3	410 (36.2)	128 (33.8)	282 (37.5)	68.8	62.1-74.7	1.24	0.86-1.79	0.249
≥ 4	456 (40.4)	164 (43.4)	292 (38.8)	64.0	58.2-69.4	1.00	-	
**Has a health card**								<0.001
No card	90 (8.0)	80 (21.3)	10 (1.4)	11.6	5.4-23.3	1.00	-	
Yes seen	840 (74.5)	135 (35.8)	705 (93.8)	84.0	80.0-87.3	39.93	17.01-93.72	<0.001
Yes not seen	197 (17.5)	161 (42.9)	36 (4.8)	18.4	12.6-26.0	1.72	0.63-4.64	0.287

Table S6: Tests for interaction terms between each variable and time.

Variable	LRT	DF	*p*-value
Province	27.2	10	**0.0024**
Urban	7.62	1	**0.0058**
Mothe's age	2.61	2	0.2720
Religion	18.6	3	**0.0003**
Antenatal visits	12.3	2	**0.0022**
Place of delivery	1.88	1	0.1700
Marital status	4.24	2	0.1200
Mother education	6.04	2	**0.0488**
Mother occupation	4.24	1	**0.0396**
Household wealth index	12.6	4	**0.0136**
Child birth order	14.1	2	**0.0009**
Sex of the child	0.05	1	0.8310

LRT – likelihood ratio test statistic (a twice the difference between the log-likelihood of the null model and the model that includes the variable)
DF – degrees of freedom
Each interaction term interaction was introduced on model 1 separately. Interaction terms with p-value below 0.10 are considered significant.

Table S7: Mixed-effect logistic regression of fully immunised children with linear time interactions, 1997-2015.

	Main Terms*			ROR*		
Characteristic	OR	95%CI	*p*-value	OR	95%CI	*p*-value
Total included (N)	4322					
**Mothers age group**						
15-24	1.00	-		1.00	-	
25-34	1.27	0.90 - 1.80	0.177	0.99	0.96 - 1.01	0.317
≥ 35	1.09	0.64 - 1.84	0.754	1.01	0.96 - 1.05	0.718
**Mothers education**						
Illiterate	1.00	-		1.00	-	
Primary	1.49	1.07 - 2.06	0.017	1.00	0.97 - 1.02	0.734
Secondary/above	2.35	0.96 - 5.77	0.061	0.99	0.92 - 1.05	0.683
**Mothers marital status**						
Single/Never in union	0.90	0.46 - 1.76	0.750	1.00	0.95 - 1.05	0.898
Married/Cohabitation	1.00	-		1.00	-	
Divorced/Separated/Widowed	1.15	0.70 - 1.91	0.576	0.97	0.93 - 1.01	0.102
**Mothers occupation**						

Unemployed	1.00	-		1.00	-	
Employed	0.89	0.63 - 1.26	0.504	1.01	0.98 - 1.04	0.477
**Area of residence**						
Urban	1.37	0.86 - 2.18	0.191	0.98	0.95 - 1.02	0.431
Rural	1.00	-		1.00	-	
**Wealth index**						
Poorest	1.00	-		1.00	-	
Poorer	1.09	0.68 - 1.75	0.726	0.98	0.95 - 1.02	0.397
Middle	1.71	1.08 - 2.70	0.022	0.99	0.95 - 1.03	0.544
Richer	1.41	0.85 - 2.34	0.178	1.01	0.96 - 1.05	0.788
Richest	2.12	1.08 - 4.15	0.029	0.96	0.91 - 1.02	0.221
**Religion**						
Catholic	1.00	-		1.00	-	
Islamic	0.66	0.41 - 1.08	0.099	1.06	1.01 - 1.10	0.009
Protestant	0.66	0.42 - 1.02	0.062	1.02	0.98 - 1.06	0.251
Others	0.77	0.48 - 1.25	0.293	1.00	0.96 - 1.04	0.865
**Antenatal visits**						
No visits	1.00	-		1.00	-	
1 to 3	6.93	4.01 - 11.97	< 0.001	0.94	0.89 - 0.98	0.009
≥ 4	8.67	5.02 - 14.97	< 0.001	0.93	0.89 - 0.98	0.004
**Place of delivery**						
At home/other	1.00	-		1.00	-	
Institutional	1.46	1.04 - 2.05	0.029	1.01	0.98 - 1.04	0.496

**Sex of the child**						
Male	1.08	0.81 - 1.44	0.590	1.00	0.98 - 1.02	0.993
Female	1.00	-		1.00	-	
**Birth order**						
1	1.00	-		1.00	-	
2 to 3	0.49	0.28 - 0.84	0.010	1.05	1.01 - 1.10	0.019
≥ 4	0.33	0.19 - 0.58	< 0.001	1.07	1.03 - 1.12	0.002
Time since 1997 (years)	1.02	0.95 - 1.10	0.548	-	-	
**Province****						
Niassa	0.62	0.37 - 1.05	0.076	1.03	0.99 - 1.08	0.162
Cabo Delgado	0.70	0.40 - 1.21	0.198	1.03	0.99 - 1.09	0.174
Nampula	0.58	0.36 - 0.94	0.026	0.99	0.96 - 1.04	0.782
Zambézia	0.51	0.31 - 0.86	0.011	1.00	0.96 - 1.04	0.996
Tete	1.10	0.68 - 1.78	0.697	0.97	0.94 - 1.01	0.190
Manica	0.75	0.47 - 1.19	0.223	0.99	0.95 - 1.02	0.480
Sofala	1.10	0.71 - 1.72	0.669	1.01	0.97 - 1.05	0.699
Inhambane	2.30	1.36 - 3.91	0.002	0.96	0.92 - 1.01	0.088
Gaza	1.73	1.03 - 2.91	0.039	1.00	0.96 - 1.04	0.985
Maputo Província	1.48	0.78 - 2.79	0.227	1.04	0.98 - 1.10	0.222
Intercept	0.34	0.14 - 0.80	0.014			
Random-Intercept SD†	0.5077					

OR – Odds ratio

ROR – Ratio of odds ratio. The ROR is time interaction term. It represents the multiplicative change of the OR per year for one particular covariate. The change is linear in the log-odds scale.
* The main terms can be viewed as the association on 1997 whereas the ROR (time interaction terms) are an average relative change of the association per year
† The SD is in logit scale. This for the PSU
** Province is coded as sum-contrasts dummy indicator. Therefore, the reference is overall Mozambique. And the coefficients here are deviations from that overall

Table S8: Multivariable logistic regression of full immunised children aged 12-23 months in 1997 to 2015 surveys.

Characteristic	Full immunisation											
	1997			2003			2011			2015		
	OR	95% CI	*p*-value	OR	95% CI	*p*-value	OR	95% CI	*p*-value	OR	95% CI	*p*-value
Total included	1080			1559			2192			1070		
**Mother's age group**			0.073			0.916			0.906			0.231
15-24	1.00	-		1.00	-		1.00	-		1.00	-	
25-34	0.79	0.41-1.57	0.514	0.91	0.59-1.41	0.682	1.03	0.69-155	0.890	1.34	0.78-2.31	0.288
≥ 35	1.55	0.57-4.25	0.393	0.94	0.55-1.59	0.815	0.95	0.58-1.55	0.831	1.85	0.92-3.74	0.087
**Mother's education**			0.013			0.001			0.783			0.004
Illitarate	1.00	-		1.00	-		1.00	-		1.00	-	
Primary	1.87	1.23-2.84	0.004	1.63	1.20-2.23	0.002	1.09	0.82-1.44	0.549	1.58	0.98-2.54	0.060
Secondary/above	7.32	0.66-81.28	0.104	8.03	1.48-43.49	0.016	0.99	0.64-1.56	0.989	3.21	1.62-6.35	0.001
**Mother's marital status**			0.827			0.753			0.974			0.296
Single/Never in union	0.99	0.33-2.92	0.981	0.77	0.38-1.54	0.452	0.99	0.59-1.67	0.974	0.83	0.38-1.81	0.632
Married/Cohabitation	1.00	-		1.00	-		1.00	-		1.00	-	

Divorced/Separated/Widowed	0.74	0.29-1.93	0.540	0.99	0.63-1.56	0.963	1.05	0.69-1.57	0.824	0.71	0.46-1.09	0.120
**Mother's occupation**			0.121			0.007			0.247			0.785
Unemployed	1.00	-		1.00	-		1.00	-		1.00	-	
Employed	0.73	0.49-1.09	0.121	0.54	0.35-0.84	0.007	1.16	0.90-1.50	0.247	1.05	0.75-1.47	0.785
**Area of residence**			<0.001			0.348			0.058			0.289
Urban	3.78	2.08-6.87	<0.001	0.79	0.49-1.28	0.348	1.38	0.99-1.93	0.058	0.69	0.36-1.36	0.289
Rural	1.00	-		1.00	-		1.00	-		1.00	-	
**Wealth index**			0.013			<0.001			0.212			0.002
Poorest	1.00	-		1.00	-		1.00	-		1.00	-	
Poorer	0.83	0.21-3.37	0.794	0.93	0.59-1.46	0.755	1.08	0.77-1.52	0.664	0.76	0.45-1.29	0.307
Middle	1.12	0.39-3.17	0.837	1.64	1.07-2.54	0.025	1.26	0.86-1.83	0.236	1.23	0.69-2.18	0.467
Richer	2.71	0.96-7.64	0.059	2.24	1.44-3.47	<0.001	1.52	1.04-2.22	0.031	2.47	1.29-4.72	0.007
Richest	1.46	0.40-5.31	0.565	4.14	2.09-8.20	<0.001	1.45	0.90-2.32	0.123	2.87	1.27-6.49	0.012
**Religion**			0.584			0.004			0.090			0.005
Catholic	1.00	-		1.00	-		1.00	-		1.00	-	
Islamic	1.11	0.46-2.68	0.817	0.79	0.51-1.24	0.307	1.49	0.99-2.42	0.058	2.62	1.52-4.54	0.001
Protestant	1.44	0.85-2.43	0.175	1.14	0.78-1.66	0.509	1.39	0.96-2.01	0.081	1.11	0.69-1.77	0.669
Others*	1.17	0.64-2.13	0.608	2.08	1.27-3.41	0.004	1.06	0.76-1.47	0.747	1.08	0.68-1.69	0.750
**Antenatal visits**			<0.001			<0.001			<0.001			0.001
No visits	1.00	-		1.00	-		1.00	-		1.00	-	
1 to 3	17.99	7.67-42.24	<0.001	4.31	2.63-7.08	<0.001	5.52	3.45-8.83	<0.001	2.28	1.16-4.48	0.017

≥ 4	22.02	9.72-49.86	<0.001	6.22	3.88-9.96	<0.001	4.77	2.93-7.77	<0.001	3.45	1.76-6.77	<0.001
**Place of delivery**			0.993			0.271			<0.001			0.030
At home/other	1.00	-		1.00	-		1.00	-		1.00	-	
Institutional	0.99	0.54-1.86	0.993	1.24	0.84-1.84	0.271	2.06	1.56-2.73	<0.001	1.66	1.05-2.62	0.030
**Sex of the child**			0.360			0.467			0.435			0.173
Male	0.75	0.41-1.39	0.360	0.90	0.68-1.19	0.467	0.91	0.72-1.15		1.30	0.89-1.90	
Female	1.00	-		1.00	-		1.00	-	0.435	1.00	-	0.173
**Birth order**			0.342			0.635			0.689			0.331
1	1.47	0.71-3.01	0.294	1.20	0.74-1.95	0.461	1.12	0.67-1.87	0.673	1.34	0.68-2.63	0.397
2 to 3	1.56	0.85-2.88	0.150	1.20	0.81-1.79	0.358	1.16	0.83-1.64	0.390	1.55	0.86-2.78	0.144
≥ 4	1.00	-		1.00	-		1.00	-		1.00	-	

Figure S1: Population pyramids of Mozambique and its provinces, using Census 2017 projections for the year 2020. [1]

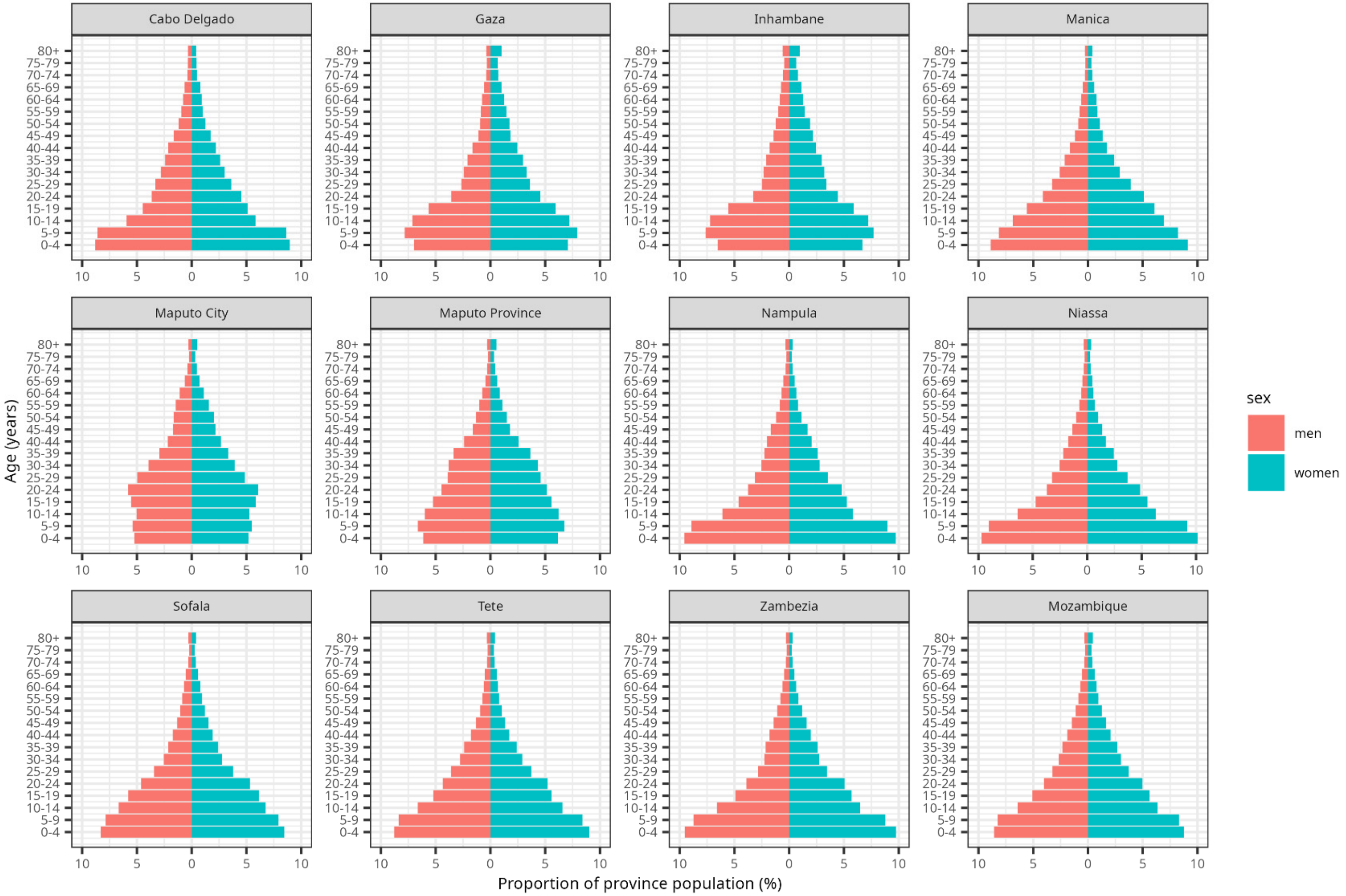

References

1 Instituto Nacional de Estatistica. INE Destaques. http://www.ine.gov.mz/ (accessed 29 Jan 2022).
